# The relative contributions of MNTB and LNTB neurons to inhibition in the medial superior olive assessed through single and paired recordings

**DOI:** 10.3389/fncir.2014.00049

**Published:** 2014-05-15

**Authors:** Michael T. Roberts, Stephanie C. Seeman, Nace L. Golding

**Affiliations:** Department of Neuroscience, Center for Learning and Memory, The University of Texas at AustinAustin, TX, USA

**Keywords:** inhibition, auditory brainstem, timing, sound localization, axon

## Abstract

The medial superior olive (MSO) senses microsecond differences in the coincidence of binaural signals, a critical cue for detecting sound location along the azimuth. An important component of this circuit is provided by inhibitory neurons of the medial and lateral nuclei of the trapezoid body (MNTB and LNTB, respectively). While MNTB neurons are fairly well described, little is known about the physiology of LNTB neurons. Using whole cell recordings from gerbil brainstem slices, we found that LNTB and MNTB neurons have similar membrane time constants and input resistances and fire brief action potentials, but only LNTB neurons fire repetitively in response to current steps. We observed that LNTB neurons receive graded excitatory and inhibitory synaptic inputs, with at least some of the latter arriving from other LNTB neurons. To address the relative timing of inhibition to the MSO from the LNTB versus the MNTB, we examined inhibitory responses to auditory nerve stimulation using a slice preparation that retains the circuitry from the auditory nerve to the MSO intact. Despite the longer physical path length of excitatory inputs driving contralateral inhibition, inhibition from both pathways arrived with similar latency and jitter. An analysis of paired whole cell recordings between MSO and MNTB neurons revealed a short and reliable delay between the action potential peak in MNTB neurons and the onset of the resulting IPSP (0.55 ± 0.01 ms, *n* = 4, mean ± SEM). Reconstructions of biocytin-labeled neurons showed that MNTB axons ranged from 580 to 858 μm in length (*n* = 4). We conclude that while both LNTB and MNTB neurons provide similarly timed inhibition to MSO neurons, the reliability of inhibition from the LNTB at higher frequencies is more constrained relative to that from the MNTB due to differences in intrinsic properties, the strength of excitatory inputs, and the presence of feedforward inhibition.

## INTRODUCTION

**FIGURE 1:**
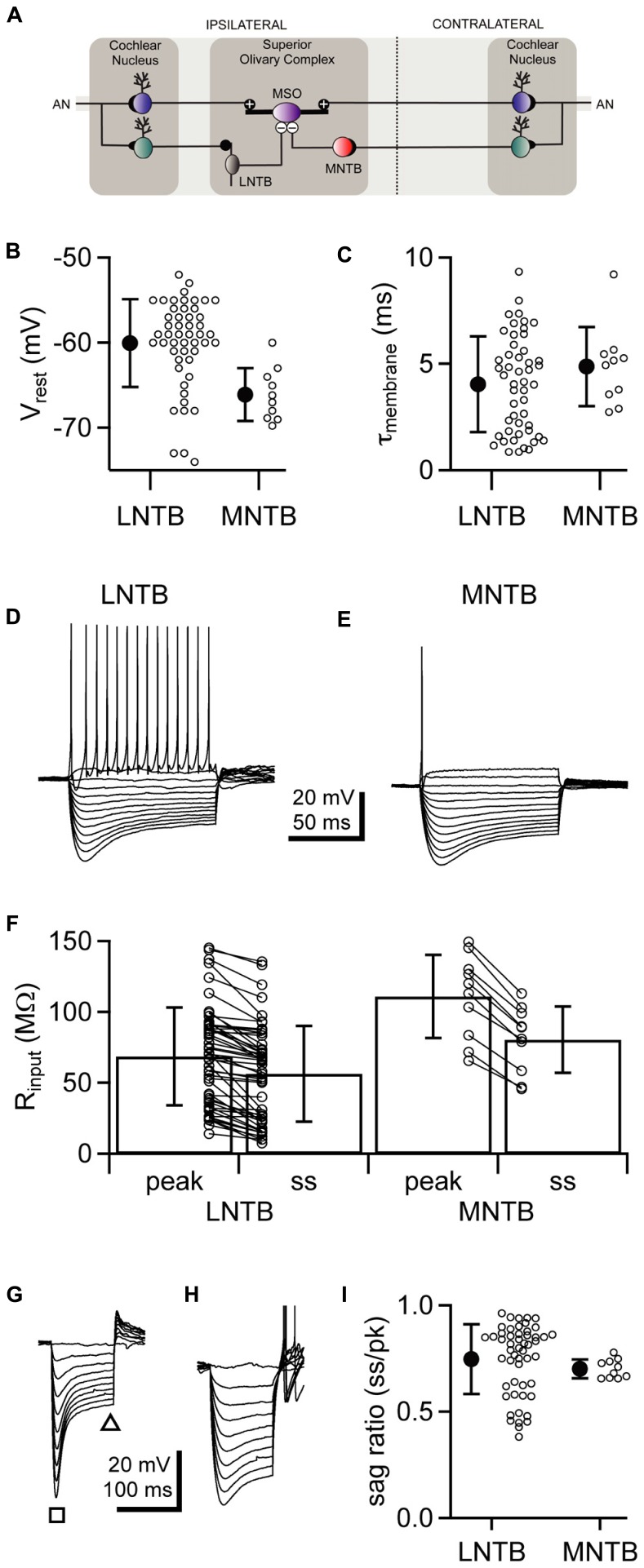
** LNTB and MNTB neurons have similar intrinsic physiology but differ in action potential firing. (A)** Cartoon illustrating circuitry from the cochlear nuclei, through the lateral and medial nuclei of the trapezoid body, to the medial superior olive. **(B,C)** Comparison of resting membrane potentials **(B)** and membrane time constants **(C)** between LNTB and MNTB neurons. **(D,E)** Responses of an LNTB **(D)** and an MNTB **(E)** neuron to a series of current steps. A suprathreshold depolarizing current step evoked repetitive firing in the LNTB neuron but only a single action potential in the MNTB neuron. **(F)** Comparison of input resistances between LNTB and MNTB neurons. Input resistances were measured at the peak and steady-state portions of the responses to a series of hyperpolarizing current steps. **(G,H)** Responses of LNTB neurons to hyperpolarizing current steps highlight the variability in *I*_h_ expression across LNTB neurons [**(G)**,sag ratio = 0.46; **(H)**, sag ratio = 0.85]. Note the presence of rebound spikes in **(H)**. The square and triangle in **(G)** indicate where peak and steady-state voltage measurements were taken, respectively. **(I)** Sag ratios measured when the peak response reached ~-80 mV show the range of *I*_h_ expression in LNTB and MNTB neurons. Open circles indicate measurements from individual cells. Error bars represent SD. *n* = 50 LNTB neurons, *n* = 10 MNTB neurons.

To identify the origin of low frequency sounds in the azimuthal plane, animals discern microsecond-order differences in the arrival times of sounds at the two ears. In mammals, neurons in the medial superior olive (MSO) detect these interaural time differences (ITDs) by comparing the timing of excitatory inputs received from pathways that begin at the ipsilateral and contralateral cochlea (**Figure 1A**; Joris and Yin, 2007; Grothe et al., 2010). *In vivo* studies, however, have long found signs that MSO computations are also influenced by inhibition (Goldberg and Brown, 1969; Yin and Chan, 1990; Spitzer and Semple, 1995). Some studies have suggested that this influence extends to defining the temporal window for coincidence detection in the MSO (Brand et al., 2002; Pecka et al., 2008), although others have concluded that coincidence detection requires only the information provided by excitatory inputs (Day and Semple, 2011; van der Heijden et al., 2013).

The main sources of inhibitory input to the MSO are neurons in the lateral nucleus of the trapezoid body (LNTB; Cant and Hyson, 1992; Kuwabara and Zook, 1992; Spirou and Berrebi, 1996) and the medial nucleus of the trapezoid body (MNTB; Spangler et al., 1985; Banks and Smith, 1992). LNTB and MNTB neurons are glycinergic (Adams and Mugnaini, 1990; Spirou and Berrebi, 1997), and receive excitatory input from globular bushy cells in the ipsilateral and contralateral cochlear nuclei, respectively (**Figure 1A**; Tolbert et al., 1982; Friauf and Ostwald, 1988; Kuwabara et al., 1991; Smith et al., 1991; Thompson and Schofield, 2000). In the *in vitro* slice preparation, stimulation of LNTB or MNTB inputs elicits fast inhibitory responses in MSO neurons (Grothe and Sanes, 1993, 1994; Magnusson et al., 2005; Chirila et al., 2007; Couchman et al., 2010; Fischl et al., 2012).

Given the importance of timing in MSO computations, the speed and temporal precision of LNTB and MNTB neurons are critical to understanding the role of inhibition in ITD detection. It is well established that bushy cell inputs to the LNTB and MNTB preserve timing information with a high degree of precision (Spirou et al., 1990; Smith et al., 1991; Joris et al., 1994a, b; Mc Laughlin et al., 2008; Rhode, 2008; Lorteije et al., 2009; Recio-Spinoso, 2012). Recently, we showed in the cochlear nucleus-superior olive (CN-SO) slice preparation that stimulation of either the ipsilateral or contralateral auditory nerve evokes inhibitory postsynaptic potentials (IPSPs) in MSO neurons that arrive earlier than excitatory postsynaptic potentials (EPSPs; Roberts et al., 2013). This suggests that the inhibitory pathways to the MSO are adapted for speed. Consistent with this, MNTB neurons receive powerful excitatory drive from the calyx of Held and have intrinsic physiology that allows rapid, reliable, and temporally precise firing (Kopp-Scheinpflug et al., 2011; Borst and Soria van Hoeve, 2012). However, direct recordings between synaptically coupled MNTB and MSO neurons have not been previously reported, leaving the timing and strength of MNTB inhibition of MSO neurons unresolved. In contrast to the MNTB, only a few studies have examined the physiology of LNTB neurons, and these have focused on responses to sound *in vivo*. These studies found that neurons in and around the LNTB are driven with short latencies by ipsilateral sounds and exhibit a variety of firing patterns (Guinan et al., 1972a, b; Tsuchitani, 1977). Thus, the physiology of LNTB neurons remains largely unexplored, leaving a large gap in our understanding of how ipsilateral inhibition to the MSO is shaped.

In the present study, we hypothesized that the intrinsic and synaptic physiology of both LNTB and MNTB neurons are adapted to provide rapid and temporally precise inhibition to the MSO. With whole cell current clamp recordings from LNTB and MNTB neurons in acute brainstem slices from the Mongolian gerbil, we found that LNTB neurons share many properties with MNTB neurons, including the ability to fire high frequency trains of action potentials. We identified an inhibitory input to LNTB neurons that may derive from other LNTB neurons, suggesting a feedforward or lateral inhibitory circuit within the LNTB. Using the CN-SO slice preparation, we found that stimulation of the ipsilateral and contralateral auditory nerves elicited IPSPs in MSO neurons with similar latencies and very low jitter. Finally, recordings from synaptically coupled pairs of MNTB and MSO neurons showed that MNTB neurons provide rapid and temporally precise inhibition to the MSO. Based on computer reconstructions of MNTB neuron axons together with synaptic latency measurements, we estimate the conduction velocities along this projection to the MSO.

## MATERIALS AND METHODS

### SLICE ELECTROPHYSIOLOGY

All procedures were conducted in accordance with National Institutes of Health guidelines and were approved by The University of Texas at Austin IACUC. Mongolian gerbils (*Meriones unguiculatus*) were deeply anesthetized with halothane or isoflurane, then their brains were rapidly removed into 32°C artificial cerebrospinal fluid (ACSF). The brainstem was isolated and transferred to a Vibratome (Leica VT1000S or VT1200S) where 200 μm-thick slices were cut in the coronal plane for LNTB recordings or the horizontal plane for MNTB and MSO recordings. Slices were incubated in 35°C ACSF for 30–60 min, then stored at room temperature until use. ACSF was continuously bubbled with 95% O_2_/5% CO_2_ and was comprised of 125 mM NaCl, 25 mM glucose, 25 mM NaHCO_3_, 2.5 mM KCl, 1.25 mM NaH_2_PO_4_, 2.0 mM CaCl_2_, and 1.0 mM MgSO_4_.

Whole cell current clamp recordings were made using a Dagan BVC-700A or Molecular Devices MultiClamp 700B amplifier. Slices were perfused with ACSF at 1–2 ml/min and visualized using differential interference contrast optics. Experiments were conducted at 35°C except for recordings between MNTB–MSO pairs, which were conducted at 37°C. Recording electrodes (2–6 MΩ) were filled with an intracellular solution comprised of 115 mM K-gluconate, 4.42 mM KCl, 0.5 mM EGTA, 10 mM HEPES, 10 mM Na_2_Phosphocreatine, 4 mM MgATP, 0.3 mM NaGTP, and 0.1% biocytin, osmolality adjusted to 300 mmol/kg with sucrose, pH adjusted to 7.30 with KOH. LNTB and MNTB recordings were made from P18–22 gerbils and MNTB–MSO paired recordings from P15–16 gerbils. Data were sampled at 50–100 kHz and lowpass filtered at 3–10 kHz. Bridge balance was compensated during all experiments. Membrane potentials are corrected for a 10 mV junction potential. Four cells in the MNTB data set were included in a previous study (Scott et al., 2005). Rheobase was found by injecting a series of 100 ms depolarizing current steps and identifying the smallest amplitude current step that elicited an action potential.

### THE CN-SO SLICE

The cochlear nucleus-superior olive (CN-SO) slice preparation was prepared as previously described (Roberts et al., 2013). In brief, coronal slices from P15–20 gerbils were cut at a thickness of 1.0–1.5 mm to preserve the circuitry from the cut end of the auditory nerve through the cochlear nuclei to the superior olivary complex. Slices were incubated in a custom interface chamber to improve oxygenation. During recordings, slices were perfused with 35°C ACSF at 8–10 ml/min. The ACSF for CN-SO slice experiments was the same as described above except that it contained 1.5 mM CaCl_2_ and 1.5 mM MgSO_4_. Stimuli to the auditory nerve stumps were delivered through suction electrodes (0.8–1.0 mm tip diameter). Whole cell current clamp recordings were made from MSO neurons visualized near the surface of the slice. IPSP onset was defined as the time at which the IPSP amplitude exceeded three times the standard deviation of the immediately preceding baseline.

### IDENTIFYING MNTB–MSO PAIRS

After patching onto an MSO neuron, a puffer pipette containing a glutamate-based solution and connected to a picospritzer (Toohey Company) was placed over the adjacent MNTB. The puffing solution contained 10 mM glutamate, 125 mM NaCl, 2.5 mM KCl, 3 mM HEPES, and 0.1% fast green for visualization. The effective spread of the glutamate-based solution was controlled by varying puff duration. To coarsely survey the MNTB for candidate presynaptic neurons, 300 ms puffs (8 psi) were applied as the puffer was moved over the MNTB in a grid-like pattern. When a puff elicited a short latency train of IPSPs in the MSO neuron, the duration of the puff was systematically decreased and the search area refined. Finally, when puff duration was decreased to 3 ms, the spatial resolution of the puffer was sufficient to discriminate between two neighboring MNTB cells (somata <200 μm apart), such that puffing onto one cell body elicited IPSPs while puffing onto the other did not. The puffer pipette was then retracted away from the surface of the slice and the candidate MNTB neuron was patched with a third pipette. If action potentials evoked in the MNTB neuron with brief current steps elicited IPSPs in the MSO neuron, the pair was synaptically coupled.

### ANATOMY

Cells were filled with biocytin via the recording electrode. Immediately after recording, slices were fixed in 4% formaldehyde in PBS and stored at 4°C. Tissue was treated with an avidin–biotin horseradish peroxidase complex (Vectastain Elite ABC kit, Vector Labs), stained with a nickel-enhanced DAB reaction, and mounted onto slides with Mowiol 4-88 (Calbiochem). Neurons were reconstructed with a Neurolucida system (MBF Bioscience) using a 100× oil immersion objective. Axonal path length, branching, and synaptic contacts were quantified using Neurolucida Explorer (MBF Bioscience).

### DATA ANALYSIS

Data were analyzed using custom algorithms implemented in IgorPro. Action potential threshold was defined as the membrane potential at which the second time derivative of the membrane potential crossed 500 mV/ms^2^. Inter-spike intervals were measured based on the time at which each spike crossed threshold. Spontaneous IPSPs were detected using a template-based algorithm (Clements and Bekkers, 1997). In brief, this method involved generating a template by fitting a bi-exponential function to a typical sIPSP. The template was then scanned across the data from a single cell, and the ability of the template to fit each consecutive set of data points equal in length to the template was assessed. When the quality of the fit exceeded a set criterion, the underlying data was considered to contain a sIPSP. Detected sIPSPs were verified by visual inspection then individually analyzed to assess sIPSP kinetics. Synaptic jitter was defined as the standard deviation of the IPSP latency for an individual cell. Mean jitter represents the average across the jitter measured for each cell in a group. Significant differences (*p* < 0.05) were detected with Student’s *t*-tests. Data are expressed as mean ± SEM unless otherwise indicated.

## RESULTS

### INTRINSIC PHYSIOLOGY OF LNTB AND MNTB NEURONS

We hypothesized that LNTB neurons, like MNTB neurons, are specialized for providing rapid and reliable inhibition to the MSO (see circuitry in **Figure 1A**). To test this hypothesis, we investigated the intrinsic physiology of LNTB neurons and compared it to that of MNTB neurons. Whole cell current clamp recordings of LNTB neurons were made in coronal brainstem slices from P18–22 gerbils. The LNTB was readily identifiable under brightfield microscopy as a lightly myelinated region located along the ventro-lateral edge of the slice. Neurons were filled via the recording pipette with biocytin, and all recovered neurons (*n* = 21) were confirmed as being in the LNTB. Whole cell current clamp recordings of MNTB neurons were made in horizontal brainstem slices from P19–22 gerbils.

We found that the resting membrane potential of LNTB neurons was depolarized relative to that of MNTB neurons on average (mean ± SD: LNTB, -60.0 ± 5.2 mV versus MNTB, -66.1 ± 3.1 mV; *p* < 0.001; **Figure 1B**), although the resting potential of some LNTB neurons was more negative than the most negative MNTB neurons in our data set. Membrane time constants, which were assessed by fitting an exponential function to the rising phase of the response to small (<3 mV) hyperpolarizing current steps, did not significantly differ between LNTB and MNTB neurons (mean ± SD: LNTB, 4.04 ± 2.26 ms versus MNTB, 4.88 ± 1.87 ms; *p* = 0.23; **Figure 1C**). In response to hyperpolarizing current steps, LNTB and MNTB neurons exhibited a depolarizing sag characteristic of the expression of hyperpolarization-activated current (*I*_h_), while depolarizing current steps elicited repetitive firing in LNTB neurons but only one to three action potentials in MNTB neurons (**Figures 1D,E**). Repetitive firing in LNTB neurons was characterized by a longer interspike interval between the first and second action potentials in a train than between subsequent action potential pairs. Action potentials in many LNTB neurons also exhibited multi-component afterhyperpolarizations. Properties of action potential firing are addressed in more detail below.

Input resistance was measured based on a linear fit to the peak and steady-state responses to current steps that hyperpolarized the membrane potential <15 mV. Both peak and steady-state input resistances were significantly lower in LNTB neurons than MNTB neurons (mean ± SD: LNTB, *R*_pk_ = 68.8 ± 34.6 MΩ, *R*_ss_ = 56.5 ± 33.7 MΩ; MNTB, *R*_pk_ = 111.0 ± 29.4 MΩ, *R*ss = 80.6 ± 23.4 MΩ; *R*_pk_, *p* < 0.01; *R*_ss_, *p* < 0.05; **Figure 1F**). Within each neuron type, the steady-state input resistance was significantly less than the peak input resistance (pairwise *t*-test: LNTB, *p* < 0.001; MNTB, *p* < 0.001). This is consistent with the expression of *I*_h_ in both neuron types. Across LNTB neurons, we noticed that the amount of sag in response to hyperpolarizing current steps could be quite large (**Figure 1G**) or more moderate (**Figure 1H**). To quantify the amount of sag, we measured the ratio of the steady-state to peak membrane potential responses to current steps that hyperpolarized the membrane potential to ~-80 mV at the peak. These sag ratios revealed that LNTB and MNTB neurons consistently expressed *I*_h_, but that the amount of expression varied considerably within the population of LNTB neurons (mean ± SD: LNTB, 0.75 ± 0.16; MNTB, 0.70 ± 0.04; *p* = 0.10; **Figure 1I**). The heterogeneity of intrinsic physiology across LNTB neurons suggests that different subsets of LNTB neurons may serve different computational roles.

**FIGURE 2:**
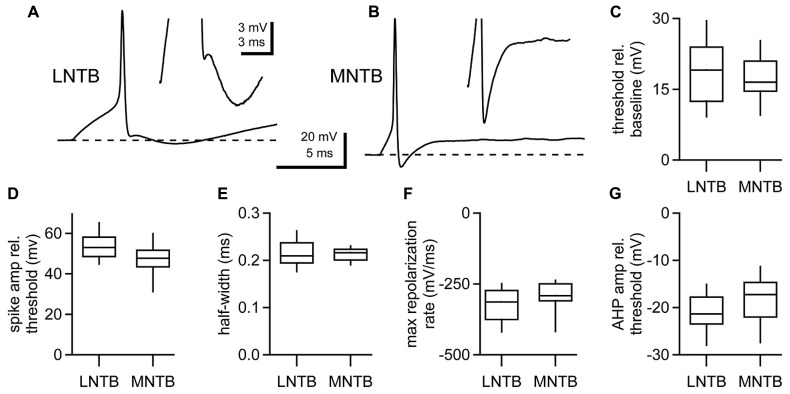
**Action potential properties assessed at rheobase in LNTB and MNTB neurons. (A)** An action potential from an LNTB neuron exhibiting an afterhyperpolarization with a double undershoot. **(B)** An action potential from an MNTB neuron with a characteristic monophasic afterhyperpolarization. **(C**–**G)** Box plots comparing properties of the first action potential fired at rheobase in LNTB and MNTB neurons. Action potential amplitudes were significantly larger in LNTB neurons (**D**; *p* = 0.03), but there was no significant difference in action potential threshold **(C)**, half-width **(E)**, maximum repolarization rate **(F)**, or afterhyperpolarization amplitude **(G)** between LNTB and MNTB neurons (*p* > 0.05). Boxes mark median, 25th and 75th percentiles. Whiskers indicate 10th and 90th percentiles. *n* = 52 LNTB neurons, *n* = 10 MNTB neurons.

To compare the properties of action potentials in LNTB and MNTB neurons, we examined the first action potential fired at rheobase, the most negative membrane potential at which a spike was evoked for a given cell. Both LNTB (**Figure 2A**) and MNTB (**Figure 2B**) neurons fired large amplitude action potentials that were brief in duration. Action potentials in MNTB neurons were followed by afterhyperpolarizations exhibiting a single repolarizing phase (**Figure 2B** *inset*). In contrast, in 65% of LNTB neurons the afterhyperpolarization was comprised of multiple phases: an initial rapid hyperpolarization followed after a brief delay by a second, slower hyperpolarization (**Figure 2A** *inset*). This type of multi-phasic afterhyperpolarization was termed a double undershoot when it was observed in some cochlear nucleus and dorsal nucleus of the lateral lemniscus neurons, and we adopt that terminology here (Oertel et al., 1990; Zhang and Oertel, 1993a, b; Wu and Kelly, 1995). The remaining LNTB neurons had simple afterhyperpolarizations similar to those in MNTB neurons. Besides the presence of double undershoots in the majority of LNTB neurons, the properties of action potentials in LNTB and MNTB neurons were largely similar. Action potential thresholds (**Figure 2C**) were well matched between the two cell types, while action potential amplitudes relative to threshold were on average 15% larger in LNTB neurons (*p* = 0.03; **Figure 2D**). Action potential half-widths were quite brief (**Figure 2E**) due in large part to a rapid rate of repolarization (**Figure 2F**) following the action potential peak. Despite differences in afterhyperpolarization shape, the most negative voltage reached during the afterhyperpolarization did not differ significantly between LNTB and MNTB neurons (**Figure 2G**). Together, the similarity in action potential properties between LNTB and MNTB neurons, particularly the brief duration and strong repolarization rate, suggests that LNTB neurons may share the capacity of MNTB neurons to fire at high frequencies.

**FIGURE 3:**
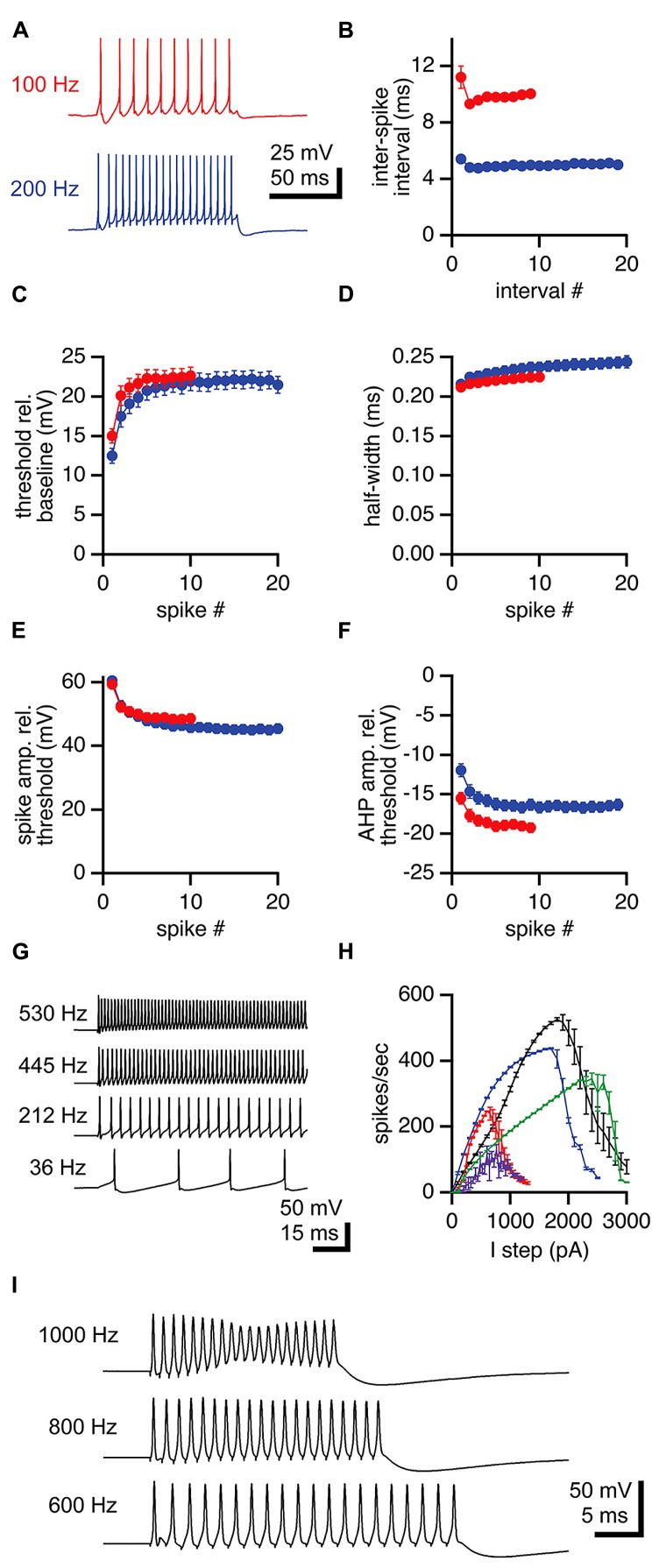
**Repetitive firing in LNTB neurons in response to current steps. (A)** An LNTB neuron responded to 100 ms current steps of 400 and 900 pA with 100 Hz (red) and 200 Hz (blue) trains of action potentials. **(B)** The first inter-spike interval was longer than subsequent intervals during trains. During sustained firing, LNTB neurons did not exhibit spike frequency adaptation. **(C)** Action potential threshold was lowest at the onset of trains, but remained relatively stable after the third action potential. **(D)** Action potential half-widths remained brief throughout trains. **(E)** Action potential amplitudes were consistently large during trains. **(F)** Afterhyperpolarization amplitudes decreased during the early portion of trains. **(B**–**F)** 100 Hz trains (red), *n* = 49. 200 Hz trains (blue), *n* = 41. **(G)** In response to 1 second current steps of (from bottom to top) 100, 700, 1300, and 1900 pA, an LNTB neuron fired trains of action potentials at frequencies of 36–530 Hz. **(H)** Action potential firing versus current injection relationships for 5 LNTB neurons. Action potential counts indicate the average number of action potentials fired during 1 s current steps. **(I)** Action potentials elicited by trains of 20 current pulses delivered at 600 Hz (1 ms, 3000 pA pulses), 800 Hz (0.5 ms, 4000 pA pulses), or 1000 Hz (0.5 ms, 4500 pA pulses). Error bars indicate SEM.

Unlike MNTB neurons, LNTB neurons fire repetitively in response to depolarizing current steps (**Figures 1D,E**). To assess the properties of action potentials in LNTB neurons during repetitive firing we analyzed responses to 100 ms current steps that elicited firing at overall rates of approximately 100 and 200 Hz (**Figure 3A**). Almost all LNTB neurons (94%) could sustain 100 Hz firing in response to current steps while most (79%) could sustain 200 Hz firing. During repetitive firing at both frequencies, the interval between the first and second action potentials was significantly longer than the interval between the second and third action potentials (paired *t*-test: 100 Hz, *p* = 0.03; 200 Hz, *p* = 0.01; **Figure 3B**). By the third or fourth pair of action potentials, the interspike interval stabilized, indicating a lack of spike frequency adaptation. Similarly, action potential thresholds increased after the first spike in the train but stabilized by the third or fourth spike (**Figure 3C**). Action potential half-widths showed a slight trend toward lengthening (**Figure 3D**) and peak amplitudes a slight trend toward decreasing (**Figure 3E**) across the 100 and 200 Hz trains. The amplitudes of afterhyperpolarizations, measured relative to the threshold of the preceding spike, became more negative following the first two to three spikes in a train, then stabilized for the remainder of the train (**Figure 3F**). As a group, these results show that the properties of action potentials in LNTB neurons are quite stable following the onset phase of repetitive firing. In five LNTB neurons, we explored the dynamic range of repetitive firing by eliciting action potentials with 1 s current steps over a range of amplitudes. While three neurons sustained firing in excess of 300 Hz, with one reaching 530 Hz (**Figure 3G**), the remaining two neurons exhibited more moderate firing rates. Thus, there is a diversity in input-output functions for LNTB neurons (**Figure 3H**).

Given the rapid repolarization rates and brief half-widths of action potentials in LNTB neurons, we suspected that at least some of these neurons might be able to fire at even higher rates than revealed by current steps. It has been shown that globular bushy cells, which provide excitatory drive to LNTB neurons, can phase lock to frequencies >1 kHz (Spirou et al., 1990; Smith et al., 1991; Joris et al., 1994a, b; Rhode, 2008; Recio-Spinoso, 2012). Thus, we injected trains of twenty brief current pulses into LNTB neurons to test their ability to phase lock to high frequency inputs. In five neurons, trains of 1 ms current pulses with amplitudes up to 2000 pA elicited firing with 96.1 ± 0.1% (mean ± SD) reliability at up to 600 Hz. In one of these neurons, we also injected trains of 20, 0.5 ms current pulses with amplitudes up to 4500 pA and observed firing at 1 kHz with 99.5% reliability (spikes/20 current pulses in 20 trials; **Figure 3I**). These results suggest that LNTB neurons *in vivo* might be capable of phase locking to bushy cell inputs at frequencies of 600 Hz or higher.

### LNTB NEURONS RECEIVE EXCITATORY AND INHIBITORY SYNAPTIC INPUTS

**FIGURE 4:**
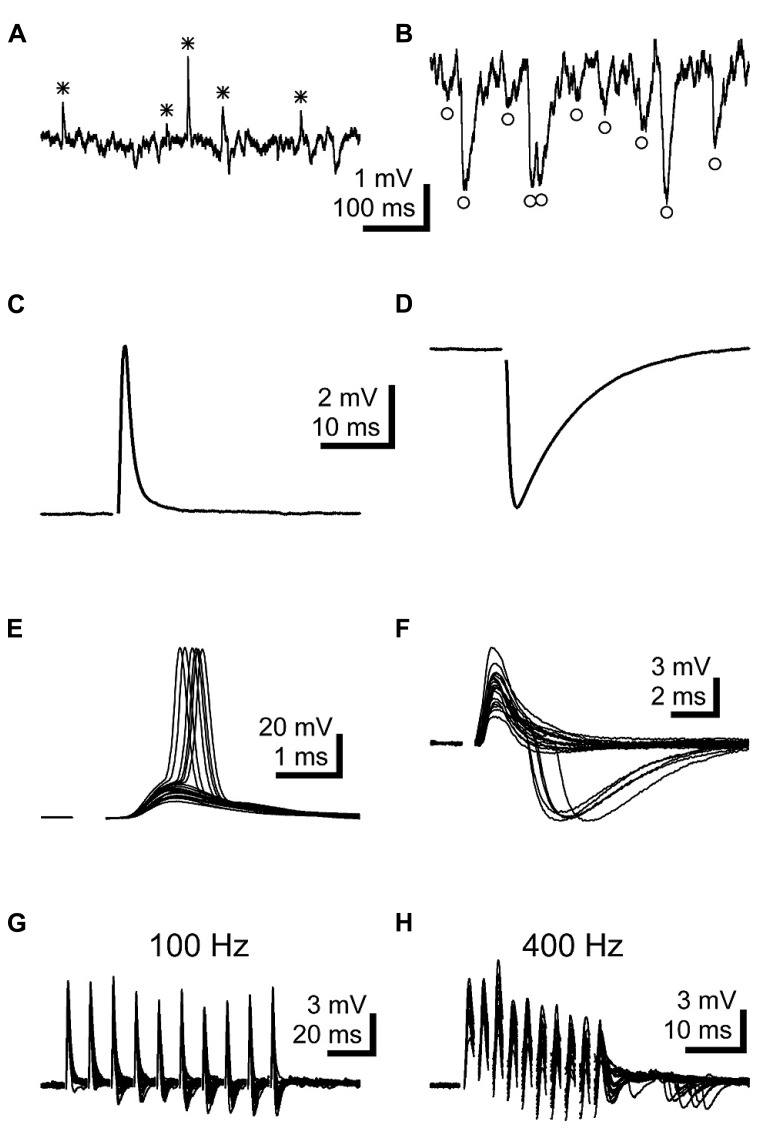
**LNTB neurons receive EPSPs and glycinergic IPSPs**. **(A,B)** Spontaneous EPSPs (A*) and IPSPs **(B°)** were commonly observed in LNTB neurons. **(C)** An EPSP evoked in an LNTB neuron from a shock delivered in the trapezoid body dorsal to the LNTB. **(D)** An IPSP evoked in an LNTB neuron with a shock delivered in the LNTB. **(E)** Overlay of 20 responses to shocks evoking EPSPs. In 6 trials, the EPSPs elicited action potentials. **(F)** Overlay of 20 responses to shocks evoking EPSPs and, in 5 trials, delayed IPSPs. The delayed onsets of the IPSPs suggest that these were elicited through a disynaptic pathway. **(G,H)** Trains of ten stimuli at 100 **(G)** and 400 Hz **(H)** evoked EPSPs and delayed onset IPSPs. IPSPs became more prominent as the trains progressed, and at 400 Hz IPSPs continued after the train offset. Overlays of 20 trials are shown.

During the course of recording from LNTB neurons, we frequently detected spontaneous EPSPs (**Figure 4A**) and IPSPs (**Figure 4B**). To more closely examine the properties of synaptic inputs to LNTB neurons, we used stimulating electrodes to directly evoke EPSPs (**Figure 4C**) and IPSPs (**Figure 4D**). Stimulus-evoked EPSPs had submillisecond rise times and half-widths of ~3 ms (**Table 1**). Stimulus-evoked and spontaneous IPSPs had much slower kinetics, with half-widths averaging ~4.5 and 8 ms, respectively (**Table 1**). Evoked IPSPs were completely blocked by 1 μM strychnine in 5 out of 5 cells, indicating that they were mediated by glycine receptors. In some neurons, evoked EPSPs were sufficiently large to evoke action potentials (**Figure 4E**). In other neurons, we noted that evoked EPSPs could be followed after a delay by an IPSP (**Figure 4F**). This delay suggests that inhibition onto LNTB neurons might involve a feedforward or lateral circuit. Consistent with this, in two LNTB neurons we found that IPSPs appeared during trains of stimuli, with a tendency to become stronger later in the train (**Figures 4G,H**). In one case, IPSPs followed the offset of the train by ~10 ms (**Figure 4H**). The role of inhibition in the LNTB is unknown, but its presence suggests that the LNTB is more than a sign-inverting relay for bushy cell inputs.

**Table 1:** Properties of EPSPs and IPSPs in LNTB neurons.

	*n*	Mean amp. (mV)	10–90% rise time (ms)	Decay τ (ms)	Half-width (ms)
Evoked EPSP	6	6.04 ± 0.92	0.56 ± 0.10	3.84 ± 2.30	3.02 ± 1.26
Evoked IPSP	6	3.44 ± 0.60	0.94 ± 0.10	4.34 ± 1.05	4.54 ± 0.81
Spont. IPSP	8	1.47 ± 0.51	2.05 ± 0.74	13.80 ± 3.47	7.96 ± 2.01

*Mean ± SEM.*

### ANATOMY OF LNTB NEURONS

**FIGURE 5:**
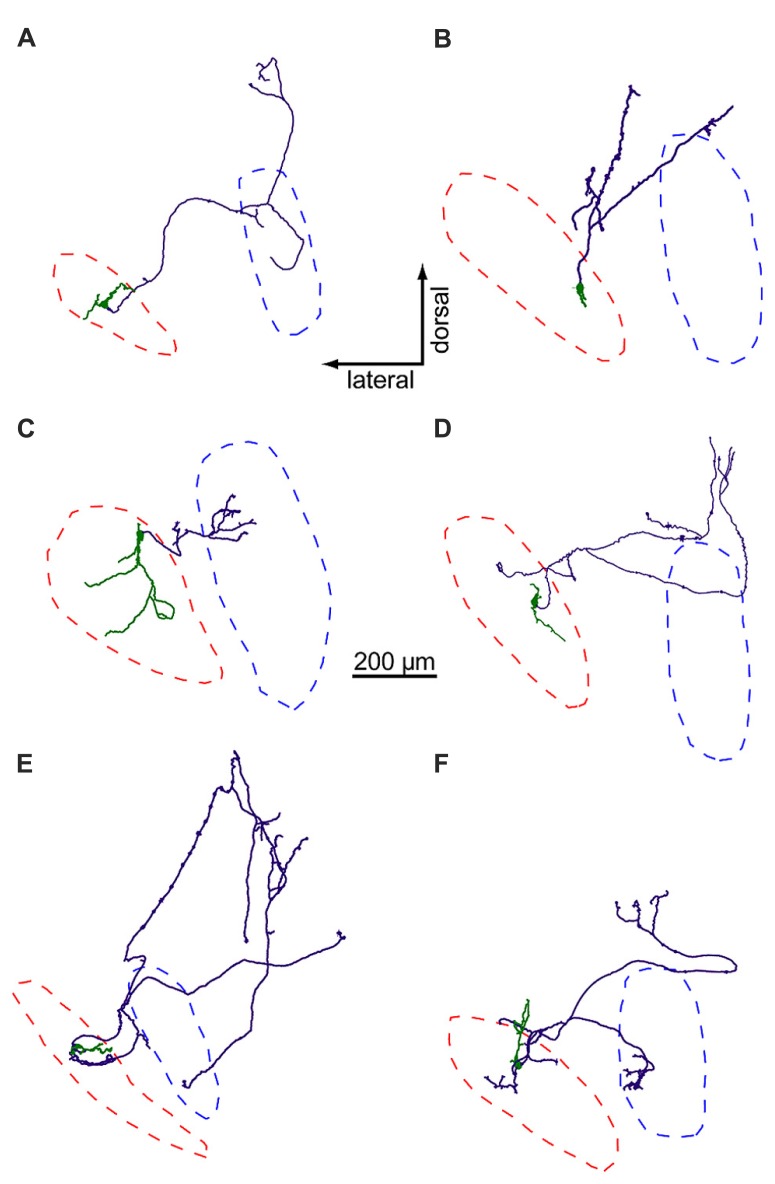
**LNTB neurons have multi-polar dendrites and axons that project to the MSO and/or periolivary nuclei**. Somata and dendrites are shown in green, axons in purple. Red and blue dashed lines mark the predicted borders of the LNTB and MSO, respectively. The axons of neurons in **(A,C,E,F)** form terminals in the MSO, while the axons in **(B,D)** do not.

Using biocytin staining, we successfully recovered anatomy for 21 of 44 neurons in the LNTB data set. Of these, six LNTB neurons possessed axons that remained in the plane of the slice (**Figure 5**). These axons exited the LNTB heading in a dorsomedial direction and branched extensively, forming a number of collaterals. In four of the neurons, one or more axon collaterals terminated in the MSO (**Figures 5A,C,E,F**). Some axons formed terminals in a region dorsal to the MSO, possibly synapsing onto neurons in the superior periolivary nucleus (e.g., **Figures 5A,F**). Interestingly, several neurons possessed axon collaterals that projected back into the LNTB, where they formed terminal fields (**Figures 5D-F**). This suggests that at least some of the inhibitory input we observed in LNTB neurons (e.g., **Figures 4F–H**) came from other LNTB neurons. Among the axons that projected to the MSO, there was a surprising diversity in the path lengths traversed prior to terminating in the MSO. Measured from the axon hillock to the terminal, three neurons possessed collaterals that followed relatively direct paths to the MSO, with average path lengths ranging from 412 to 678 μm (mean ± SD: 544 ± 133 μm). The fourth neuron possessed an axon collateral that followed a very indirect path, traveling 978 μm dorsomedially before turning back to terminate in the ventral portion of the MSO, 1917 μm away from the axon hillock (**Figure 5E**). It is unclear whether the axonal projection pattern of this neuron represents an unusual case or if it is typical of a subset of LNTB neurons.

The dendritic arbors of LNTB neurons provide another example of morphological diversity. Some neurons possessed relatively simple dendritic arbors with little branching (**Figures 5A,B,D,E**), while others possessed larger arbors with more extensive branching (**Figures 5C,F**). The dendrites of some neurons mostly extended along an axis parallel to the long axis of the LNTB (**Figures 5B,D**), while others extended orthogonally (**Figures 5A,E**). The dendritic arbor of one neuron extended to cover much of the LNTB (**Figure 5C**). At present, there is little information about the *in vivo* tuning curves of LNTB neurons. Based on the diversity in dendritic arbor morphology shown here, we predict that some LNTB neurons may receive bushy cell inputs representing a narrow band of sound frequencies while others may be much more broadly tuned.

### TIMING OF INHIBITION TO THE MSO

Studies of MNTB neurons have shown that the coupling between presynaptic input and postsynaptic spiking is highly reliable, rapid, and temporally precise (Mc Laughlin et al., 2008; Lorteije et al., 2009). Based on *in vivo* recordings in the MSO, it has also been proposed that the timing of inhibition provided by MNTB neurons to the MSO (i.e., contralaterally driven inhibition) is sufficiently precise that sound driven inhibition can arrive prior to the arrival of sound driven excitation (Brand et al., 2002; Pecka et al., 2008). Previously, we developed a novel slice preparation that retains the circuitry from the auditory nerve through the cochlear nucleus to the superior olivary complex (Roberts et al., 2013). This cochlear nucleus-superior olive (CN-SO) slice preparation allowed us to directly stimulate the auditory nerve while using whole cell current clamp recordings to assess responses elicited in MSO neurons. These experiments provided direct evidence that stimulation of either the ipsilateral or contralateral auditory nerve elicited IPSPs in MSO neurons that, on average, arrived 0.32 ± 0.13 ms (ipsilateral) or 0.38 ± 0.09 ms (contralateral) prior to the arrival of EPSPs. Here, we performed additional analysis of this data set to test the hypothesis that nerve-driven inhibition to the MSO is as fast and temporally precise along the ipsilateral (LNTB) pathway as it is widely assumed to be along the contralateral (MNTB) pathway.

**FIGURE 6:**
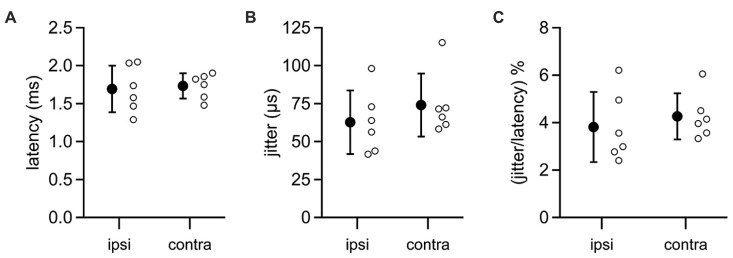
**Latencies from auditory nerve stimuli to IPSPs in MSO neurons**. Recordings were made from MSO neurons in the CN-SO slice preparation, which retains circuitry from the auditory nerve to the MSO. IPSPs were evoked by direct stimulation of the auditory nerve stump on the ipsilateral or contralateral side of the slice. Neither the latency to IPSP onset **(A)** or the jitter in IPSP latencies **(B)** differed significantly between ipsilateral and contralateral pathways (*t*-tests: latency, *p* = 0.79; jitter, *p* = 0.37). **(C)** The jitter in IPSP timing represented a small percentage of the total latency to IPSP onset (*t*-test, *p* = 0.55). Open circles denote data from individual MSO neurons. Error bars show SD. Ipsilateral, *n* = 6; contralateral, *n* = 6.

In five MSO neurons, we were able to evoke IPSPs by directly stimulating the ipsilateral auditory nerve. Ipsilateral IPSPs arrived with a latency of 1.69 ± 0.31 ms (**Figure 6A**) and jitter of 0.06 ± 0.02 ms (mean ± SD; jitter = SD of latency for an individual cell; **Figure 6B**). In five other neurons, IPSPs were elicited by stimulating the contralateral auditory nerve. These data showed that the latency to contralateral IPSPs (mean ± SD: 1.73 ± 0.17; **Figure 6A**) was not significantly different from the latency to ipsilateral IPSPs (*t*-test: *p* = 0.79), nor was there a significant difference between the contralateral jitter (mean ± SD: 0.07 ± 0.02 ms) and the ipsilateral jitter (*t*-test: *p* = 0.37; **Figure 6B**). Signals along both the ipsilateral and contralateral inhibitory pathways must traverse three synapses and two cell types to travel from the auditory nerve to the MSO. Given this, it is remarkable that the jitter along the ipsilateral and contralateral pathways is less than 5% of the total latency to IPSPs (mean ± SD: ipsi, 3.81 ± 1.47%; contra, 4.26 ± 0.97%; *p* = 0.55; **Figure 6C**). These results indicate that LNTB neurons in the ipsilateral inhibitory pathway can at least transiently provide inhibition to the MSO with the same high speed and precision as provided by MNTB neurons in the contralateral inhibitory pathway.

**FIGURE 7:**
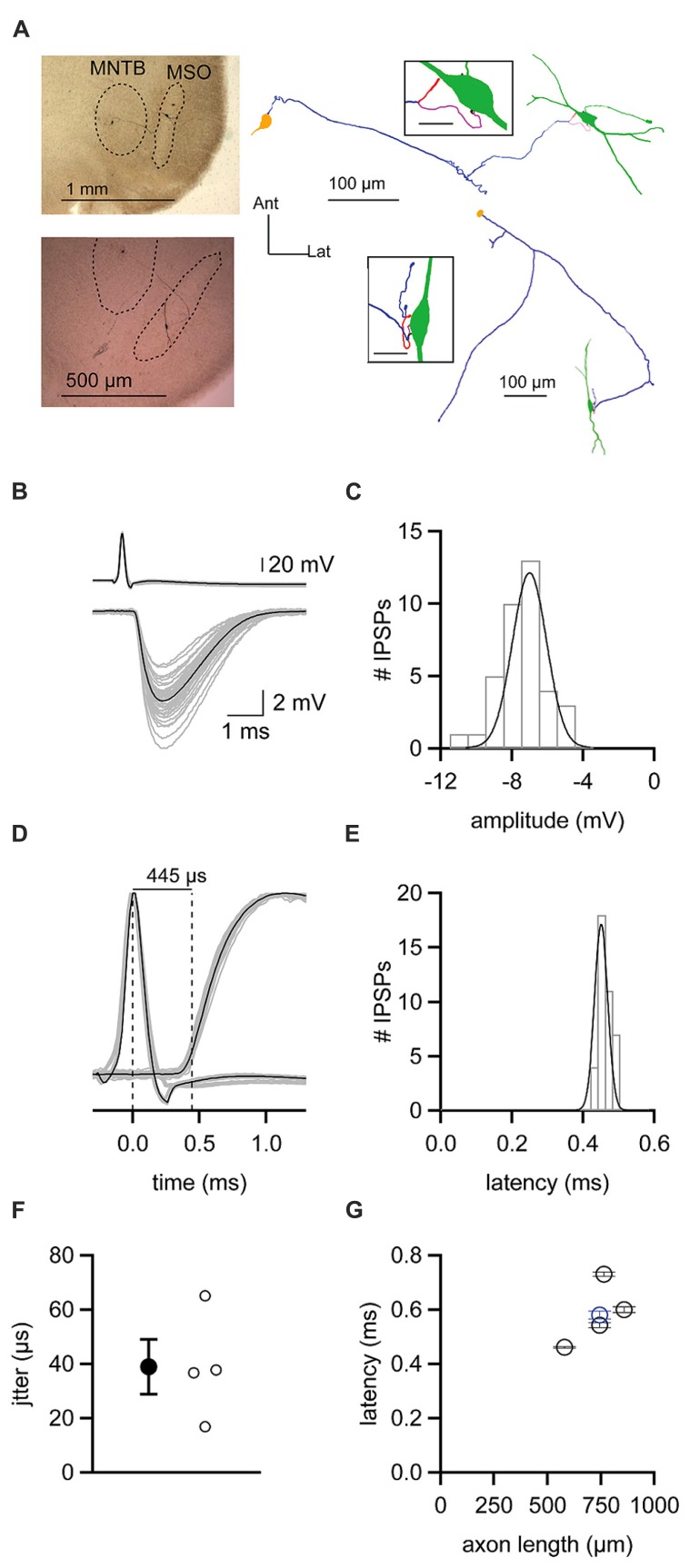
**Latencies from action potentials in MNTB neurons to IPSPs in MSO neurons (A)** Left, micrographs showing staining of biocytin-filled, synaptically coupled MNTB and MSO neuron pairs. Right, reconstructions of the pairs shown on the left. MNTB somata are yellow, axons are purple. MSO neurons are green. Insets show putative synaptic contacts (magenta) between MNTB neuron terminals and MSO neurons. Inset scale bars = 20 μm. **(B)** Action potentials elicited in an MNTB neuron (top) lead to IPSPs in a postsynaptic MSO neuron (bottom). Gray data show 38 individual trials. Black data shows the average of these trials. **(C)** Histogram of IPSP amplitudes observed from the cell shown in **(B)**. Data are fit with a Gaussian function. **(D)** Plot of data shown in **(B)** with action potential and IPSP amplitudes normalized to reveal the average 445 μs latency between the peak of the presynaptic action potential and the 20% rise of postsynaptic IPSP. (**E**) Histogram of latency measurements for individual IPSPs from the cell shown in **(B**–**D)**. Data are fit with a Gaussian function. **(F)** Mean (filled circle) and individual (open circles) jitters in the onset of IPSPs from four MNTB–MSO pairs. **(G)** Relationship between the latency to IPSP onset and the length of the axon connecting the MNTB neuron to the MSO neuron soma. Black data are from four pairs recorded at 37°C. Blue data point is from one pair recorded at 35°C. Error bars show SEM.

The finding that ipsilateral and contralateral inhibition is temporally well matched is surprising given the length disparity of the axons of the respective presynaptic excitatory inputs. To more directly assess the speed at which inhibition provided by the MNTB reaches the MSO, we recorded from synaptically coupled pairs of MNTB and MSO neurons. Such pairs were identified by patching onto an MSO neuron and using glutamate puffs to search the MNTB for potential presynaptic partners. When a small glutamate puff onto a visualized MNTB neuron evoked an IPSP in the MSO neuron, we patched onto the MNTB neuron. Synaptic coupling was confirmed if an action potential elicited by a brief current step in the MNTB neuron (**Figure 7B** top) was followed after a brief delay by an IPSP in the MSO neuron (**Figure 7B** bottom). IPSPs elicited in this way could be rather large (mean ± SEM: 6.94 ± 0.23 mV; **Figure 7C**), but averaged 3.90 ± 2.52 mV (mean ± SD) across the five synaptically coupled pairs we obtained. These pairs allowed us to measure the latency from the peak of an evoked action potential in an MNTB neuron to the 20% rise of the IPSP in the MSO neuron (**Figures 7D,E**). For the example cell shown in **Figures 7B-E**, the latency was 0.46 ms with a jitter of 0.02 ms. Across the five pairs in the data set, the latency averaged 0.58 ± 0.04 ms and the jitter 0.04 ± 0.01 ms (mean ± SEM; **Figure 7F**). For each pair, we successfully recovered the anatomy of the MNTB and MSO neuron (**Figure 7A**). Previous studies showed that glycinergic inhibition to the MSO undergoes a developmental shift from a uniform somatodendritic distribution to one that is biased strongly to the soma (Clark, 1969; Kapfer et al., 2002; Couchman et al., 2010, 2012). In agreement with these findings, we could visualize 1-4 putative synaptic contacts, with most located on the soma or nearby on the proximal dendrites (**Figure 7A** insets). These numbers can only be considered a minimum estimate because the black biocytin labeling of the cell obscured visualization of all contacts except those on the sides of the cell body. Reconstructions of recovered neurons revealed axon lengths from the MNTB to the MSO ranging from 580 to 858 μm and averaging 738 ± 45 μm (mean ± SEM). There was a correlation between the latency to the IPSP recorded in the MSO and axon length (*r* = 0.63; **Figure 7G**). Paired recordings between the calyx of Held and MNTB neurons have shown that the time required for synaptic transmission is ~0.2–0.3 ms at 35°C (Fedchyshyn and Wang, 2007). Assuming a synaptic delay in this range, our measurements of axon lengths and IPSP latencies predict that the axonal conduction velocity for MNTB neurons was ~1.9–2.6 m/s.

**FIGURE 8:**
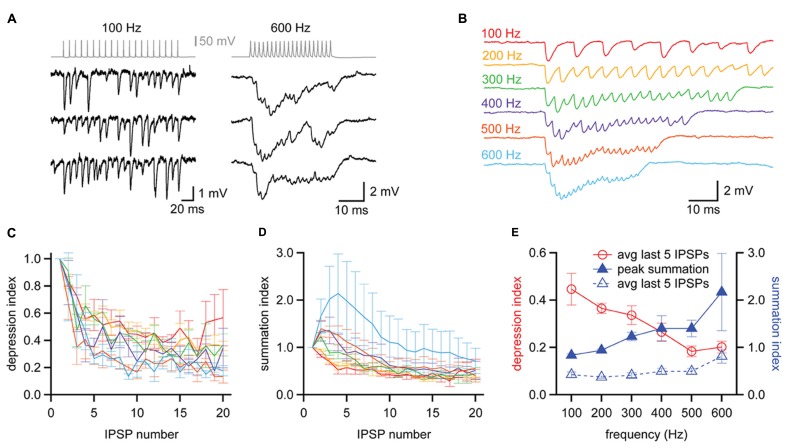
**Balanced synaptic depression and temporal summation of MNTB to MSO IPSPs during high frequency trains. (A)** Trains of IPSPs observed in an MSO neuron (black data, bottom) in response to trains of 20 action potentials evoked at 100 and 600 Hz in an MNTB neuron (gray data, top). Three trials at each frequency are shown for the MSO neuron. **(B)** Average responses of the MSO neuron in **(A)** to trains of action potentials elicited at 100–600 Hz in the presynaptic MNTB neuron. *n* = 7 trials at each frequency. **(C)** The depression index reports the foot-to-peak amplitudes of each IPSP relative to the amplitude of the first IPSP in the train, revealing that depression occurred at all frequencies tested. **(D)** The summation index reports the baseline-to-peak amplitude of each IPSP as a function of the amplitude of the first IPSP in the train. **(C,D)** Colors follow the scheme in **(B)**. **(E)** The average depression index across the last 5 IPSPs of trains (red data, open circles) decreased with increasing train frequency while the peak summation index of trains (blue data, closed triangles) increased with increasing frequency. The average summation index across the last 5 IPSPs of trains was relatively insensitive to changes in train frequency (blue data, open triangles), suggesting that the effects of depression and summation counteract each other. *n* = 3. Error bars show SEM.

*In vivo* recordings have shown that MNTB neurons rarely fire isolated action potentials but instead fire repetitively, with spikes phase-locked to sound stimuli at rates that can exceed 500 Hz (Spirou et al., 1990; Kopp-Scheinpflug et al., 2008; Lorteije et al., 2009). We next asked how this high frequency activity in the MNTB affected the strength and temporal resolution of inhibition delivered to the MSO. In three of our synaptically coupled MNTB–MSO pairs we were able to examine the inhibition produced in the MSO by trains of twenty action potentials elicited in the MNTB neuron at frequencies ranging from 100 to 800 Hz. We found that two of the MNTB neurons were able to sustain firing at 600 Hz without failure (e.g., **Figure 8A**, top right), while the third achieved 800 Hz without failure. At the relatively low frequency of 100 Hz, MNTB spikes elicited IPSPs in the MSO that were distinct and clearly distinguishable (**Figure 8A**, left). At 600 Hz, however, IPSPs overlapped and exhibited temporal summation (**Figure 8A**, right). The average IPSP trains elicited across trials at a particular frequency revealed that IPSPs underwent short term depression at all frequencies and exhibited temporal summation early in the train at frequencies ≥300 Hz (**Figure 8B**). To quantify the amount of short term depression, we measured the foot-to-peak amplitude of each IPSP in an average train and compared it to the amplitude of the first IPSP (**Figure 8C**). This depression index showed that depression was evident throughout the entire frequency range. The average depression index across the last five IPSPs in each train was 44.6 ± 6.7% at 100 Hz and decreased to 20.2 ± 2.5% at 600 Hz (**Figure 8E**). To assess the extent of temporal summation during trains, we measured IPSP amplitudes relative to the resting membrane potential for each IPSP in an average train and compared these to the amplitude of the first IPSP (**Figure 8D**). This summation index revealed that IPSP amplitudes toward the end of trains were surprisingly similar across the frequencies tested. Indeed, the average summation index across the last five IPSPS in each train was 42.7 ± 5.7% at 100 Hz and remained stable at less than 50% until increasing at 600 Hz to 81.5 ± 14.6% (**Figure 8E**). The peak value of the summation index increased as a function of train frequency (**Figure 8E**), exceeding a value of 1 at 300 Hz and higher frequencies. This indicates that temporal summation countered the effects of short term depression at higher frequencies. Thus, the interplay between temporal summation and short-term depression allows MNTB neurons to provide relatively consistent levels of inhibition during the sustained portion of an inhibitory response, regardless of input frequency.

## DISCUSSION

The physiology and temporal precision of LNTB and MNTB neurons are key determinants of how inhibition shapes coincidence detection in the MSO. Here, we investigated how LNTB and MNTB neurons are adapted for their roles in sound localization, providing the first descriptions of the intrinsic physiology of LNTB neurons and the first recordings from synaptically coupled pairs of MNTB and MSO neurons. We found that LNTB neurons share many properties with MNTB neurons, including brief action potentials and a capacity to fire at high frequencies, but differ in their ability to fire repetitively during sustained depolarizations and the strength of their excitatory inputs. We uncovered glycinergic input to LNTB neurons, suggesting a feedforward or lateral inhibitory circuit within the LNTB. Auditory nerve stimulation in CN-SO slice recordings showed that LNTB and MNTB neurons provide inhibition to the MSO with similar latencies and jitters. With paired recordings between synaptically coupled MNTB and MSO neurons, we found that propagation along MNTB axons was extremely fast and consistent across trials, providing a possible explanation for how the contralateral inhibitory pathway matches the timing of the ipsilateral pathway despite having to traverse a much longer distance. Together, these results support the hypothesis that the LNTB and MNTB provide rapid and precise inhibition to the MSO, while also suggesting that inhibition from LNTB neurons will be less reliable during sustained stimuli.

### IMPLICATIONS OF LNTB PHYSIOLOGY AND MORPHOLOGY FOR IPSILATERAL INHIBITION OF THE MSO

In the absence of *in vivo* recordings of IPSPs in MSO neurons, the dynamics of sound-evoked inhibition in the MSO must largely be inferred from the properties of LNTB and MNTB neurons. For the MNTB, it is reasonable to assume that the fast and reliable conversion of calyx of Held input into action potentials, as evidenced by strong phase locking to auditory stimuli *in vivo*, enables MNTB neurons to provide reliable and temporally precise inhibition to the MSO (for reviews, see Kopp-Scheinpflug et al., 2011; Borst and Soria van Hoeve, 2012). Our results suggest three reasons why inhibition from LNTB neurons might be more nuanced, particularly during sustained stimuli.

First, input-output coupling in the MNTB is extremely powerful due to the calyx of Held synapse, which can release up to 100 vesicles in response to a single presynaptic spike (Borst and Sakmann, 1996) and which produces EPSCs in MNTB neurons that are generally much larger than what is required to exceed action potential threshold (Brew and Forsythe, 1995; Mc Laughlin et al., 2008; Lorteije et al., 2009; Berger et al., 2014). In contrast, we found that stimulus-evoked EPSPs in LNTB neurons could be small and were typically subthreshold. This suggests that synchronous synaptic input from multiple globular bushy cells, such as that presumably elicited by auditory nerve stimulation in the CN-SO slice experiment, might be required to bring an LNTB neuron to spike. In addition, the relatively extensive dendritic morphology of the LNTB neurons for which we recovered anatomy supports the idea that individual LNTB neurons receive input from multiple bushy cells. Previous anatomical studies have similarly described the dendrites of LNTB neurons as spanning significant portions of the width of the LNTB, and presumably representing a broad range of frequencies (Kuwabara and Zook, 1992; Kulesza, 2008).

Second, low voltage-activated Kv1 channels almost always limit the response of an MNTB neuron to one action potential per EPSP or depolarizing current step (Brew and Forsythe, 1995; Dodson et al., 2002; Klug and Trussell, 2006). This 1:1 relationship between input and output underlies the ability of MNTB neurons to accurately phase lock to a sound stimulus. In the LNTB, however, we found that while isolated EPSPs elicited single action potentials, sustained current steps evoked repetitive firing. This indicates that low voltage activated K^+^ channels in LNTB neurons can limit excitability in response to a single EPSP but are not powerful enough to do so during a sustained depolarization, such as would be expected during periods of high frequency input when EPSPs undergo temporal summation.

Third, while action potentials in MNTB neurons are followed by brief afterhyperpolarizations (Forsythe and Barnes-Davies, 1993; Brew and Forsythe, 1995), those in LNTB neurons were typically much slower and were characterized in nearly two-thirds of cases by double undershoots. The effect of these strong afterhyperpolarizations was apparent during repetitive firing in LNTB neurons, increasing interspike intervals particularly between the first two action potentials in a train (**Figure 3**). This result suggests that the conductance responsible for the slow phase of the after hyperpolarization in LNTB neurons, i.e., the second undershoot, inactivates during repetitive firing. Thus, the influence of afterhyperpolarizations on LNTB neuron excitability might vary according to the level of activity.

Despite these properties, LNTB neurons were capable of reliably sustaining firing rates of hundreds of Hz in response to sustained depolarizations, and up to 1 kHz in response to trains of brief current steps. This capacity to fire at high frequencies was aided by the brevity of action potentials in LNTB neurons, a property shared with MNTB neurons. In MNTB neurons, high voltage activated K^+^ channels belonging to the Kv3 family constrain the duration of action potentials, which promotes high frequency firing by limiting inactivation of voltage gated Na^+^ channels (Brew and Forsythe, 1995; Wang et al., 1998; Li et al., 2001; Klug and Trussell, 2006). Kv3 channels probably also underlie action potential repolarization in LNTB neurons. This similarity to MNTB neurons suggests that temporal coding is important to the function of LNTB neurons, but the graded EPSPs, less influential Kv1 channels, and long afterhyperpolarizations in LNTB neurons lead us to propose that LNTB neurons provide less consistent inhibition than MNTB neurons. In particular, we suspect that LNTB neurons will provide reliable and temporally precise inhibition during the onset phase of a sound, but that reliability and precision will degrade as a sound continues.

### THE ROLE OF INHIBITION WITHIN THE LNTB

Our observation of glycinergic IPSPs in LNTB neurons provides functional confirmation of anatomical studies showing inhibitory terminals contacting the somata of LNTB neurons (Spirou and Berrebi, 1997; Spirou et al., 1998). These studies proposed that inhibitory terminals came from neurons within the LNTB or other periolivary nuclei. Our anatomical results support this, showing that the axon collaterals of some LNTB neurons projected back into the LNTB. In addition, given the limits of the circuitry left intact in a 200 μm-thick brain slice, our findings of putatively disynaptic IPSPs (**Figure 4F**) and instances where IPSPs were increasingly elicited in LNTB neurons during trains of stimuli (**Figures 4G,H**) also support a local source of LNTB inhibition. In such a circuit arrangement inhibition in the LNTB would function in a feedforward or lateral manner, depending on whether the presynaptic neuron receives input from the same or different globular bushy cells (Roberts and Trussell, 2010). In either case, it is possible that inhibition improves temporal precision in LNTB neurons by limiting the duration of the excitation provided by an EPSP (Pouille and Scanziani, 2001). Alternatively, our train data suggest that inhibition might dampen the excitability of LNTB neurons during periods of sustained activity, such as that following the onset phase of a sound. This model is particularly compelling considering the temporal summation of IPSPs expected from their relatively slow kinetics. A similar mechanism has been proposed for the role of inhibition in the MNTB (Awatramani et al., 2004).

### TIMING OF IPSILATERAL VERSUS CONTRALATERAL INHIBITION TO THE MSO

The axons of globular bushy cells travel a greater distance to reach the MNTB than the LNTB (Spirou et al., 1990; Smith et al., 1991). Despite this, our CN-SO slice recordings found no significant difference in the latency to IPSPs evoked by stimulation of the ipsilateral versus the contralateral auditory nerves. In a previous study, within-cell comparisons of ipsilateral and contralateral EPSP latencies revealed a trend (*p* = 0.192) of ipsilateral EPSPs arriving 0.20 ms faster than contralateral EPSPs (Roberts et al., 2013). If a similarly small trend exists for IPSPs, it probably could not have been detected in our experiment, which involved between-cell comparisons of IPSP latencies. Nonetheless, there is ample evidence to suggest that the short latency to contralateral IPSPs may be attributed to mechanisms that speed transmission within the contralateral pathway. In particular, coupling between synaptic release at the calyx of Held and spiking in MNTB neurons is consistently fast and highly dependable. *In vivo* recordings from mice and cats have shown that the latency from a calyx of Held spike to a postsynaptic MNTB spike is ~0.40–0.50 ms (Mc Laughlin et al., 2008; Lorteije et al., 2009). This tight coupling requires large, rapidly-rising EPSCs (Taschenberger and von Gersdorff, 2000; Iwasaki and Takahashi, 2001; Joshi and Wang, 2002; Joshi et al., 2004; Koike-Tani et al., 2005; Yang et al., 2011). In contrast, stimulus-evoked EPSPs in LNTB neurons were graded in amplitude and often did not elicit action potentials. This suggests that spiking in LNTB neurons might require synchronous input from multiple bushy cell axons and that the timing of input-output coupling in LNTB neurons is slower than in MNTB neurons. The contralateral pathway may also be adapted for rapid transmission between the MNTB and the MSO. In our paired MNTB–MSO recordings, the mean latency from an MNTB spike to an MSO IPSP was 0.58 ms, and, based on anatomical reconstructions, we estimated the axonal conduction velocity to be 1.9–2.6 m/s. The conduction velocity of LNTB axons is currently unknown, but the wide range of axon lengths we observed for LNTB projections to MSO hint that, in some cases at least, axon conduction times may be longer for LNTB than MNTB.

The temporal measurements made here and those from previous studies provide a foundation for making predictions about the conduction time in globular bushy cell axons (Seidl et al., 2010, 2014; Seidl, 2013). In the CN-SO slice, the average latency from contralateral auditory nerve stimulation to IPSPs in the MSO was 1.73 ms. Given a 0.58 ms mean latency from an MNTB spike to an MSO IPSP and assuming a 0.40 ms latency from a presynaptic terminal spike to a postsynaptic spike for both the endbulb of Held synapse onto globular bushy cells and the calyx of Held synapse onto MNTB neurons, the remaining time for axonal conduction in the contralateral pathway is ~0.35 ms. If we then assume that the axon length from a globular bushy cell to an MNTB neuron is twice that of the same axon to an LNTB neuron, we see that the increased travel distance to the MNTB may only add ~0.17 ms to the contralateral transit, a time that might easily be compensated for by the calyx of Held synapse. Interestingly, anatomical studies have revealed calyceal synapses in the most ventral portion of cat LNTB (Stotler, 1953; Adams, 1983; Spirou and Berrebi, 1996; Spirou et al., 1998). While it is unknown whether calyceal synapses are present in the ventral LNTB of gerbil, none of the LNTB neurons that we found projecting to the MSO were located in the ventral region of the LNTB, suggesting that such synapses are not a requirement for LNTB neurons that inhibit MSO neurons.

## Conflict of Interest Statement

The authors declare that the research was conducted in the absence of any commercial or financial relationships that could be construed as a potential conflict of interest.
